# Preparation, Characterisation, and Application of Bifunctional BaSO_4_ Sheets

**DOI:** 10.3390/ma13132903

**Published:** 2020-06-28

**Authors:** Jia Luo, Jiaqi Liu, Xiaoyan Guo, Yuejiao Liu, Haibo Jin, Daidi Fan

**Affiliations:** 1 College of Chemical Engineering, Beijing Institute of Petrochemical Technology, Beijing 102617, China; 2019311037@bipt.edu.cn (J.L.); 2018520011@bipt.edu.cn (J.L.); liuyuejiao@bipt.edu.cn (Y.L.); jinhaibo@bipt.edu.cn (H.J.); 2 Beijing Key Laboratory of Fuels Cleaning and Advanced Catalytic Emission Reduction Technology, Beijing 102617, China; 3 College of Chemical Engineering, Northwest University, Xi’an 710069, China; fandaidi@nwu.edu.cn

**Keywords:** barium sulfate, cellulose acetate, bifunctional sheet, dry chemical diagnosis reagent

## Abstract

Barium sulfate (BaSO_4_) is a material with high reflectance for preparing bifunctional sheets used in dry reagent chemical tests. In this study, bifunctional BaSO_4_ sheets with scattering power and high reflectance were prepared with BaSO_4_ microspheres sized 1.3~1.8 μm and cellulose acetate (CA). Factors such as the BaSO_4_ morphology, CA dosage, mixing time, surfactant, solid content and wet sheet thickness were investigated. Scanning electron microscopy (SEM), a dynamic contact angle test, light reflection detector and light reflection densitometer were employed to characterize the structure and properties of bifunctional BaSO_4_ sheets. The optimal conditions for preparing bifunctional BaSO_4_ sheets under natural drying conditions were as follows: mass ratio of CA to BaSO_4_ microspheres was 0.03:1; mixing acetone solution was used to form liquid stock puree; 35 μL Tween-80 was used to improve the hydrophilicity of bifunctional sheets; solid content was 54.9%; mixing 2 h then 450 µm thick sheets were cast on a glass plate using a film applicator. The light reflectance value of bifunctional BaSO_4_ sheets in the range of 400~800 nm was higher than 97%. Serum diffused in bifunctional BaSO_4_ sheets reacted in reagent sheets and formed uniform colorful spots. Considering the repeatability of spot proportion and light reflectance value, the sheet offered a uniform serum scattering power and good repeatability. Therefore, the bifunctional BaSO_4_ sheets are regarded as a promising material for dry chemical diagnostic reagents.

## 1. Introduction

In vitro diagnosis can be divided into two categories: wet chemical diagnosis and dry chemical diagnosis [1]. Dry chemical diagnosis refers to a method in which the liquid in a detected sample is used as a reaction medium, and the analyte directly reacts with a solid reagent solidified on the carrier [2]. Wet chemistry diagnosis refers to a method of liquid reaction after mixing a liquid reagent with a liquid sample in the reaction vessel [2]. Compared with wet chemical diagnosis, dry chemical diagnosis has advantages of using simple instruments, easy operation, fast response, easy storage and easy transportation [3,4,5,6,7]. The dry chemical diagnosis is a multi-layer structure which is composed of a bifunctional sheet, reagent sheet and support sheet. Bifunctional sheets have scattering power and high reflectance. Glucose (GLU), high-density lipoprotein (HDL) or other ingredients to be analyzed in serum or urine can be scattered on bifunctional sheets then reacted in a reagent sheet which has a certain reagent. A support sheet is a high light transmittance material which could also support a bifunctional sheet and reagent sheet. Some studies mainly used filter paper or cellulose membrane as a diagnostic reagent serum scattering power sheet [8,9], which leads to uneven dispersion, inaccurate results and is non-suitable for clinical use [10]. Using a cellulose membrane as a scattering power sheet, because of its low reflectance and inability to preset the pre-reaction reagent, means the application range is narrow [11,12].

High-reflectance materials, such as titanium dioxide (TiO_2_) and barium sulfate (BaSO_4_) are mostly used to improve various properties of the sheet. For example, TiO_2_ improves the hydrophilicity of the sheet, and a TiO_2/_cellulose acetate (CA) sheet and a BaSO_4_/CA sheet can improve the reflectance. The light reflectance value of a TiO_2_ bifunctional sheet is less than 92% and 92~98% in the wavelength range of 400~450 nm and 450~800 nm, respectively [13], while a bifunctional sheet prepared with BaSO_4_ can reach 97% and over 98% in the range of 400~450 nm and 450~800 nm, respectively [14], which is higher than a TiO_2_ bifunctional sheet. The bifunctional sheets prepared by BaSO_4_ microspheres with narrow particle size distribution are used in dry chemical diagnosis [15], which is not only conducive to form a wider response range, but also can form uniform micron-level secondary voids between particles to filter and uniformly disperse serum. BaSO_4_ with the characteristics of low cost, abundant sources and good stability is one of the functional materials for advancement. BaSO_4_ is widely used as weighting agent [16,17], radiation protection material, catalyst carrier [16], sound insulation material, paint, adsorbent [18], medical X contrast agent [19,20], additives in polymers [21,22,23,24,25,26,27] and other applications in ceramics, pulp and paper [28], medicine, batteries, cosmetics and many other industries.

In this study, the bifunctional sheets with scattering power and high reflectance are prepared by BaSO_4_ microspheres with narrow particle size distribution. The structure and performance of the sheets are mainly characterized by scanning electron microscopy (SEM), a dynamic contact angle test, light reflection detector and light reflection densitometer.

## 2. Materials and Methods

### 2.1. Materials

BaSO_4_ microspheres (1.3~1.8 µm) were prepared in our own laboratory [14]. CA weighted 30,000 g∙mol^−1^ of average molecular was procured from Sino Pharm Group (Beijing, China). Tween-80 was procured from J&K Scientific (Beijing, China). Acetone and dehydrated ethanol procured from the Beijing reagent factory (Beijing, China) were employed as solvents. Distilled water was used as a non-solvent. Serum was employed by Ortho-clinical Diagnostics, Inc. (Rochester, NY, USA)

### 2.2. Sheet Preparation

CA solution was prepared from 0.9 g CA and 30 mL acetone. Subsequently 10 g BaSO_4_ microspheres, 35 μL Tween-80, and 10 mL CA solution were added into the mixture which was then agitated for 2 h at 350 r/min. Afterward, 450 µm thick sheets were cast on a glass plate using a film applicator. Finally, the sheets were placed in a ventilated place at 25 °C for drying naturally, then a BaSO_4_ bifunctional sheet was prepared.

TiO_2_ bifunctional sheet and BaSO_4_ bifunctional sheet were coated on laboratory-made GLU dry chemical reagent sheet respectively to achieve GLU-TiO_2_/CA and GLU- BaSO_4_/CA detection chips. BaSO_4_ bifunctional sheet was coated on a laboratory-made HDL dry chemical reagent sheet to achieve multi-layer membrane structure HDL- BaSO_4_/CA detection chip which is composed of bifunctional sheet, reagent sheet and support sheet.

### 2.3. Sheet Characterization

#### 2.3.1. Scanning Electron Microscopy (SEM)

The ZEISS-SUPRA 55 field-emission scanning electron microscope produced in Germany was employed to observe the structure of a bifunctional sheet (SUPRA-55 SEM, Zeiss, Jena, Germany) [10]. The sample to be measured was sputter-plated with a fine coating, so that the highest resolution of the emission was 0.8 nm and the acceleration voltage was 5 kV. The measurement was carried out with an SE2 detector, and the measurement distance was 6.3 mm.

#### 2.3.2. Dynamic Contact Angle Test

The wettability of the BaSO_4_ bifunctional sheet was characterized by a DSA 30 optical contact angle meter manufactured by KRUSS, Germany (DSA 30, KRUSS, Hamburg, Germany) [29]. The sample to be tested was fixed on the test platform, and droplet angle method was used, then the contact angle was read by a rapid photographic apparatus. Volume of liquid was 2 μL and rate of liquid dripping was 0.16 mL∙min^−1^.

#### 2.3.3. Determination of Solid Content

The coating liquid was prepared when optimal mass ratio of CA to BaSO_4_ was 0.03:1, where the mass of BaSO_4_ microspheres was 10 g, and the volume of solvent acetone in CA solution is 8, 10, and 12 mL, respectively. The solid content of the coating liquid at 25 °C was calculated with the following Equation (1):

(1) $$\mathrm{SC}\%=\frac{m_{S}}{m_{L}}\times 100\%$$

m_S_ and m_L_ are the solid mass (mass of BaSO_4_ microspheres) and coating liquid mass (total mass of BaSO_4_ microspheres, CA and acetone), respectively.

#### 2.3.4. Determination of Wet Sheet Thickness

According to the method mentioned above, bifunctional sheets were prepared with different wet sheet thicknesses. 10 μL of serum was added and diffused through a bifunctional sheet to form a spot; the scattering power time and the spot diameter were measured, respectively.

#### 2.3.5. Determination of Reflectance

The light reflectance value of the BaSO_4_ bifunctional sheet was measured by Lambda 950 reflectance spectrometer manufactured by Perkin Elmer, USA (Lambda 950, Perkin Elmer, Waltham, MA, USA) [10]. Scanning speed of the sample was medium speed, with incident angle as 0°.

#### 2.3.6. Particle Size Distribution Tests

The particle size was determined by Rise-2008 PSD, which manufactured by Jinan Runzhi Co., Ltd., China (Rise-2008, Jinan Runzhi Co., Ltd., Jinan, China). The real part of refractive index of particles is 1.64, with water as medium, and the real part of the refractive index is 1.33 [30].

#### 2.3.7. Determination of Scattering Power

Reagent sheet was prepared and cut into five pieces, then BaSO_4_ bifunctional sheet was placed on each reagent sheet. Five 6 μL serum samples were added onto the surface of BaSO_4_ bifunctional sheets separately, which were then batch operated at 37 °C for 5 min. When the serum diffused through bifunctional sheets and reacted in reagent sheets, colored spots finally formed. The diameters of the spots and reflection rate were determined.

The standard deviation (SD) and coefficient of variation (CV) were calculated with following Equations (2) to (4):

(2) $$\mathrm{SD}\%=\sqrt{\frac{\sum_{i=1}^{n}{\left(x_{i}-\bar{x}\right)}^{2}}{n-1}}\times 100\%\;$$

(3) $$\bar{X}=\frac{\sum X_{i}}{n}$$

(4) $$\mathrm{CV}\%=\frac{\mathrm{SD}}{\bar{X}}\times 100\%$$

## 3. Results

### 3.1. Analysis of BaSO_4_ Particles

**Figure 1:**
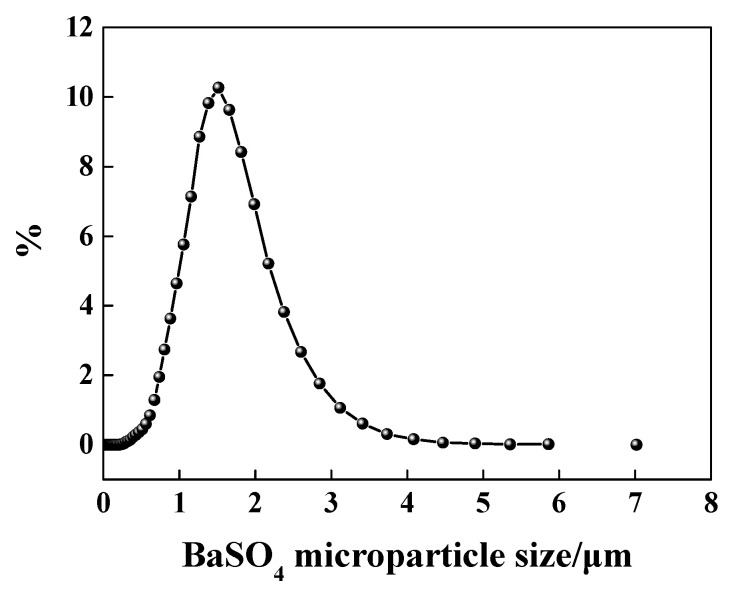
Particle size distribution of BaSO_4_ micro-particle.

The result of BaSO_4_ particle size is shown in Figure 1. As this figure shows, the average particle size was equal to 1.52 μm.

### 3.2. BaSO_4_ Morphology

**Figure 2:**
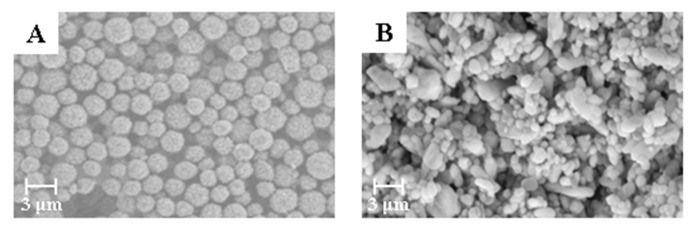
Scanning electron microscope (SEM) micrographs of BaSO_4_ bifunctional sheets with different BaSO_4_ morphology: (**A**) BaSO_4_ microspheres; (**B**) amorphous BaSO_4_.

According to the method mentioned above, commercially available amorphous BaSO_4_ powder and narrow particle size BaSO_4_ microspheres were used as raw materials to prepare bifunctional sheets. The sheets were characterized by SEM and results are shown in Figure 2.

The bifunctional sheet prepared with narrow particle size BaSO_4_ microspheres has narrow pore distribution. While the sheet prepared with commercially available amorphous BaSO_4_ powder has uneven pore distribution. This is because the particle size of commercially available amorphous BaSO_4_ powder varies widely. Also, the small particles are prone to natural agglomeration. After natural drying, the prepared bifunctional sheet is fragile due to the small binding force of particles between the natural agglomerates. Therefore, BaSO_4_ microspheres with narrow particle size were appropriate for raw materials of the bifunctional sheet’s coating.

### 3.3. Sheet Morphology

According to the method mentioned above, BaSO_4_/CA bifunctional sheets were prepared by taking CA solution in different concentrations, so the mass ratio of CA to BaSO_4_ is 0.02: 1, 0.03: 1, 0.04: 1, and 0.05: 1, respectively. When the mass ratio of CA to BaSO_4_ is 0.02: 1, the adhesion between BaSO_4_ microspheres in the bifunctional sheet is low, so it is difficult to form a whole and is easy to break. As the mass ratio increases to 0.03: 1, the adhesion is greatly improved. When the mass ratio of CA to BaSO_4_ is 0.04:1 and 0.05:1, respectively, the BaSO_4_ bifunctional sheets were formed with a uniform and smooth surface.

**Figure 3:**
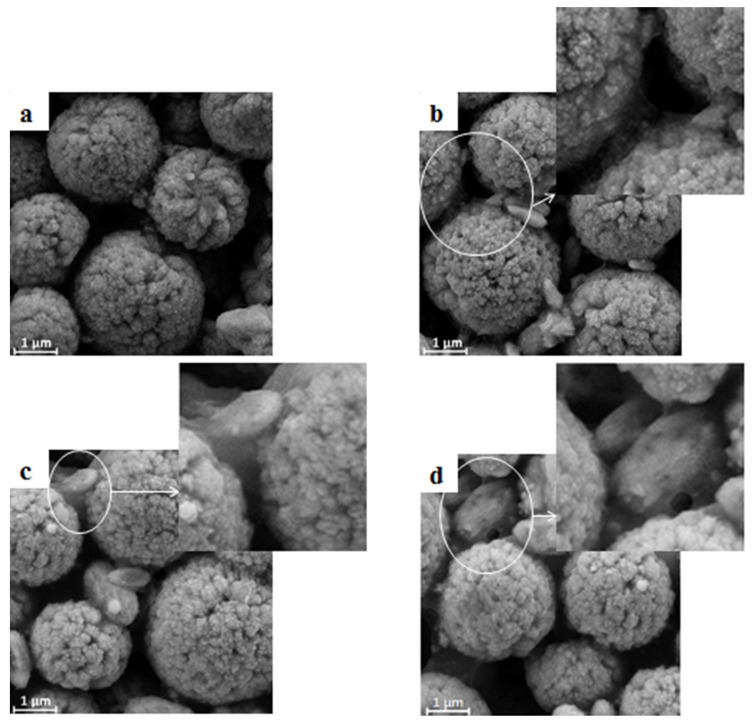
SEM micrographs of bifunctional BaSO_4_ sheets with different mass ratio of cellulose acetate (CA) to BaSO_4_: (**a**) 0.02:1; (**b**) 0.03:1; (**c**) 0.04:1; (**d**) 0.05:1.

The sheets were characterized by SEM and results are shown in Figure 3. Different mass ratio of CA to BaSO_4_ has a great influence on void distribution and void diameter in bifunctional sheets. When the mass ratio is 0.03: 1, the void distribution and void diameter are uniform (Figure 3b). As the mass ratio increases, the void diameter continues to decrease. Meanwhile, BaSO_4_ microspheres are wrapped by CA (Figure 3c–d). It can be seen from the enlarged view that the CA in connection point of particles increased while void decreased (Figure 3b–d). Because serum has a certain viscosity, the scattering power decreased obviously when void decreased.

In summary, when the mass ratio of CA to BaSO_4_ microspheres is 0.03:1, bifunctional sheets can form a monolithic sheet with uniform void diameter and uniform void distribution, which is beneficial to diffuse the sample evenly.

### 3.4. Mixing Time

**Figure 4:**
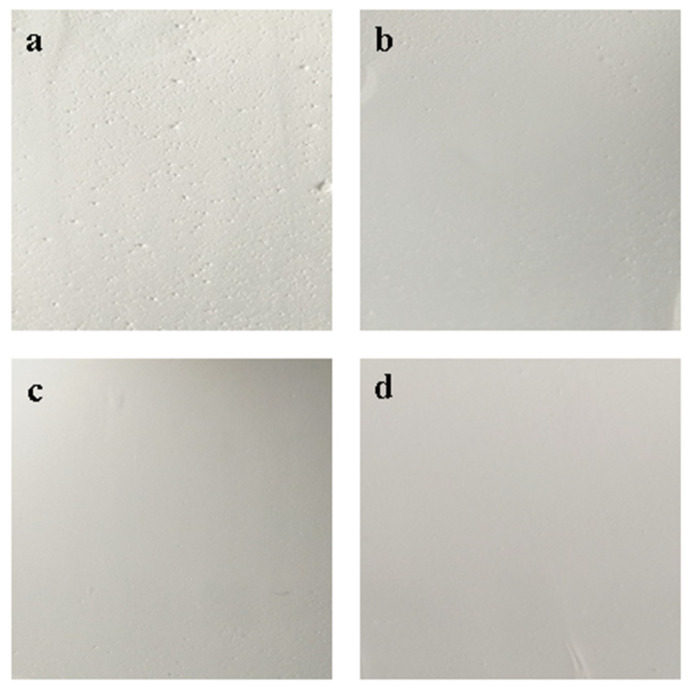
BaSO_4_ bifunctional sheets prepared with different mixing times: (**a**) 0.5 h; (**b**) 1 h; (**c**) 1.5 h; (**d**) 2 h.

BaSO_4_ bifunctional sheets prepared with a mass ratio of 0.03: 1 were prepared after different stirring times, and the results are shown in Figure 4.

With the stirring time increased, the surface of bifunctional sheets became gradually smooth because it took a certain time for CA solution to be uniformly dispersed in the slurry. Short stirring time made it difficult for CA to unevenly disperse in the slurry and wrapped around the BaSO_4_ microspheres to form larger particles, resulting in a very rough surface of bifunctional sheets. Sheets also became fragile because of the protruding particles present on the surface of bifunctional sheets. As the stirring time increases, CA solution became uniformly dispersed, and an apparent smooth surface of bifunctional sheets formed (Figure 4a–d). Therefore, to prepare bifunctional sheets with a uniform and apparent smooth surface, optimal stirring time was 2 h at room temperature.

### 3.5. Surfactant

**Figure 5:**
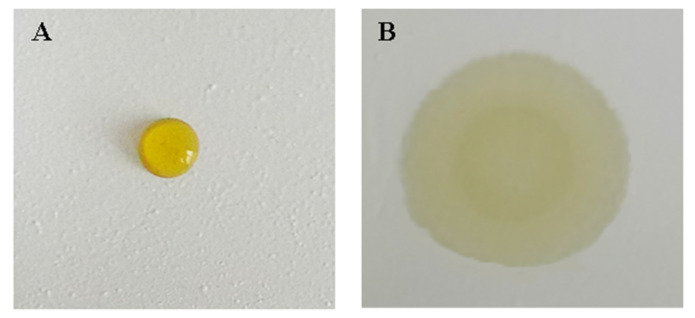
The contrast diagram of effect of surfactant on serum scattering power: (**A**) without surfactant; (**B**) with 35 μL surfactant Tween-80.

A bifunctional sheet named A with no surfactant added, and the one named B added with 35 μL surfactant Tween-80 were prepared. The results of adding 6 μL serum onto A and B, respectively, after spreading of 10 s, are shown in Figure 5.

Serum floats on A cannot diffuse to the lower reagent sheet. While serum drop on B diffused to reagent sheet quickly. This is because CA is hydrophobic. When the surface of BaSO_4_ microspheres is wrapped with CA, the bifunctional sheet is hydrophobic, so it is difficult for the serum sample to wet with the hydrophobic bifunctional BaSO_4_ sheet. Surfactant can efficiently change the degree of infiltration of the droplet on the solid surface, so the serum can easily wet the bifunctional sheet and then diffuse to the next sheet.

**Figure 6:**
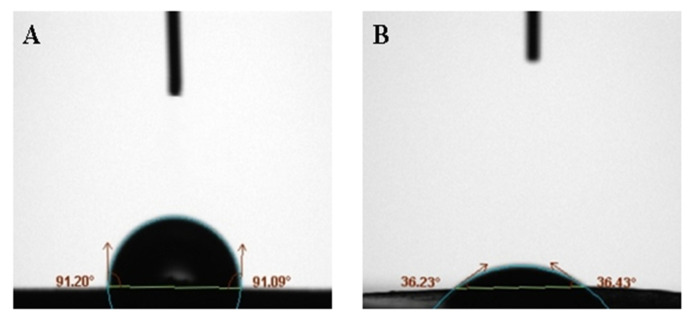
Contact angle of serum on the surface of scattering power sheets: (**A**) without surfactant; (**B**) with 35 μL surfactant Tween-80.

Figure 6 shows the contact angle between BaSO_4_ bifunctional sheets and serum prepared without adding surfactant against adding surfactant Tween-80. The contact angle of A is greater than 90°, indicating that the surface of A is highly hydrophobic, so serum easily forms a liquid bead and it is hard to wet the solid. While the contact angle of B is 36°, indicating that the surface of B is hydrophilic, so the serum easily wets the solid. Surfactant Tween-80 can be added to improve the hydrophobicity of the sheet surface and improves the scattering power of BaSO_4_ bifunctional sheet.

In the previous study, contact angles of TiO_2_ bifunctional sheet without adding surfactant and adding surfactant were 95° and 45°, respectively. This shows that the BaSO_4_ bifunctional sheet has better wettability than the TiO_2_ bifunctional sheet.

### 3.6. Solid Content

The Equation (1) was used to calculate the solid content. The influence of solid content on the surface and flatness of the bifunctional sheet was investigated, and the results are shown in Table 1.

When coating a certain area and a certain wet sheet thickness, the amount of BaSO_4_ and CA in coating liquid with a solid content of 50.5% is less than that with a solid content of 60.1%. After the wet sheet is dried, sheets with solid content of 50.5%, BaSO_4_ and CA materials are insufficient, resulting in a thin and porous sheet; when the solid content is 54.9%, a uniform and flat sheet can be formed; as the solid content increases to 60.1%, there are many solid materials, and the stirring is not uniform, resulting in the uneven surface of the bifunctional sheet. Therefore, the optimal solid content is 54.9%.

### 3.7. Wet Sheet Thickness

The influence of different wet sheet thicknesses on the scattering power of a bifunctional sheet was shown in Table 2.

When the thickness of the wet sheet is thin, the scattering power time of the serum is longer and the diameter of the formed spot is larger; as the thickness of the wet sheet is increased to 450 µm and 500 µm, the minimum scattering power time of the serum is 11 s and the minimum diameter of the spot is 1.2 cm. This is because when the thickness of wet sheet is thin, the scattering power of serum in the vertical direction is relatively short, and cannot be dispersed into enough small droplets, which makes it difficult to enter the reagent sheet, and serum will diffuse in the horizontal direction of the bifunctional sheet, forming spots with a larger diameter. Considering the cost, the wet sheet thickness of the bifunctional sheet is preferably 450 µm.

### 3.8. Reflection Tests

**Figure 7:**
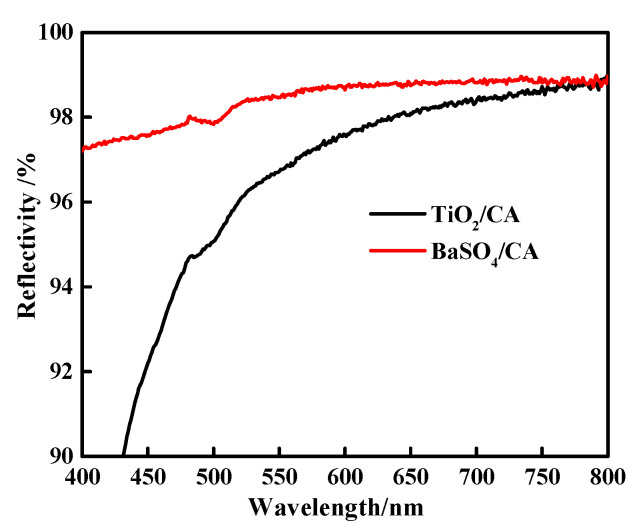
Light reflectivity of BaSO_4_ bifunctional sheet and TiO_2_ bifunctional sheet.

The light reflectance value of the BaSO_4_ bifunctional sheet was measured using a Lambda 950 reflectance spectrometer and results compared with the TiO_2_ bifunctional sheet prepared before were shown in Figure 7.

Figure 7 shows that in the wavelength range of 400~800 nm, the reflectance of the BaSO_4_ bifunctional sheet has always been superior to that of TiO_2_. The light reflectance value of the TiO_2_ bifunctional sheet is less than 92% and 92~98% in the wavelength range of 400~450 nm and 450~800 nm, respectively [7], while the bifunctional sheet prepared with BaSO_4_ can reach 97% and over 98% in the range of 400~450 nm and 450~800 nm, respectively. So, compared with TiO_2_ bifunctional sheet, the BaSO_4_ bifunctional sheet has more excellent light reflection performance and can be used for in vitro diagnostic dry chemical reagents.

### 3.9. Scattering PowerTests

Bifunctional sheets were applied to reagent sheets for measuring HDL, and the HDL dry chemical in vitro diagnostic reagent was obtained. In the reagent sheet, main reactions were as follows:

HDL + non-HDL HDL + non-HDL↓(5)

HDL Cholesterol + HDL-C + Protein(6)

HDL-C + H_2_O Cholesterol + Fatty acid (7)

Cholesterol + O_2_ 4-cholest-3-ene +H_2_O_2_ (8)

Leuco dye + H_2_O_2_ Blue dye + H_2_O (9)

**Figure 8:**
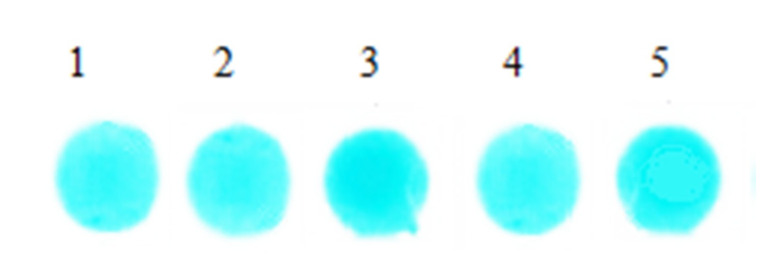
Spot images of serum reaction.

We added 6 μL serum with same concentration onto the surface of the 5 sheets, respectively, and detected at 670 nm. After reacting 5 min under 37 °C, 5 groups of reaction coloring results were obtained and are shown in Figure 8. The reflected light signal values of the 5 groups bifunctional sheets were measured by light reflection densitometer, and the results are shown in Table 3.

Figure 8 shows that after serum samples filtered and diffused through bifunctional sheets, spots color formed in reagent sheets are uniform, which indicates that the serum sample can be evenly diffused in the BaSO_4_ bifunctional sheets. In addition, the SD of 5 spots is 0.038. Table 3 is the value of the reflected light signal of the 5 spots of bifunctional sheets tested by the light reflection densitometer. The relative deviation is within the acceptable range of ±0.01%, indicating that BaSO_4_ bifunctional sheets have a narrow distribution of pore diameters not only on the surface, but also in the interior. Therefore, the scattering power of serum by BaSO_4_ bifunctional sheets is uniform and the repeatability is good.

In the previous study, the SD of TiO_2_ bifunctional sheet is 0.04, and the relative deviation of the reflected signal value is within ±0.028% [7], which indicates that the BaSO_4_ bifunctional sheet has better repeatability than the TiO_2_ bifunctional sheet.

### 3.10. Chip Detection

**Figure 9:**
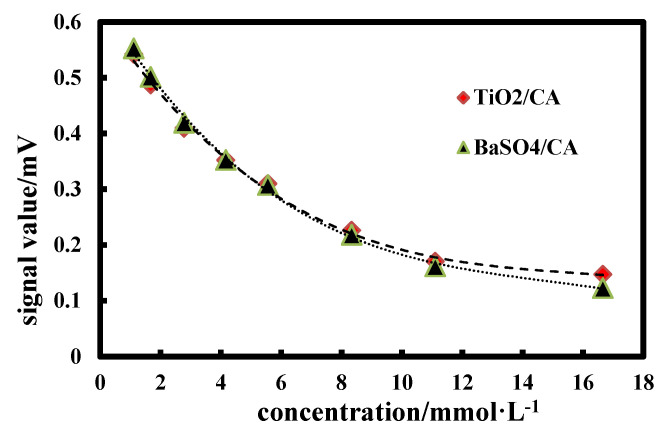
Standard curves of glucose (GLU)-TiO_2_/CA and GLU-BaSO_4_/CA detection chips at different GLU concentrations.

Figure 9 shows the standard curve of GLU-TiO_2_*/*CA and GLU-BaSO_4_*/*CA detection chips at different GLU concentrations. It can be seen that GLU-BaSO_4_*/*CA detection chip has a wider response range than GLU-TiO_2_*/*CA detection chip, especially in high concentration of 11.10~16.65 mmol∙L^−1^. Since BaSO_4_ bifunctional sheet has higher light reflectance value than the TiO_2_ bifunctional sheet (Figure 7), it can reflect more reflected light under high GLU concentration and be detected by the instrument. If less reflected light exceeds detection limit of the instrument, the signal value does not change significantly. Meanwhile, in a high concentration of 11.10~16.65 mmol∙L^−1^, the change trend of the ordinate signal value of GLU-TiO_2_/CA detection chip is not as obvious as that of GLU-BaSO_4_/CA detection chip, resulting in no obvious change in slope of the curve, which will decrease accuracy of concentration detection. So, GLU-BaSO_4_/CA detection chip has a wider response range.

### 3.11. Repeatability Tests

Serum with a concentration of 8.33 mmol L^−1^ was added to test the repeatability of two detection chips GLU-TiO_2_/CA and GLU-BaSO_4_*/*CA and the results are shown in Table 4.

Table 4 shows that the SD and CV values of GLU-TiO_2_*/*CA detection chip are 0.24 and 2.8%, respectively. While the SD and CV values of GLU-BaSO_4_*/*CA detection chip are 0.20 and 2.4%, respectively. Therefore, GLU-BaSO_4_*/*CA detection chip has better repeatability.

### 3.12. Accuracy Tests

Serum with a known concentration of 1.67 mmol·L^−1^ was determined and repeated 3 times. It can be seen from Table 5 that the accuracy results of GLU-BaSO_4_/CA detection chip are within the specified target value range, indicating that the accuracy of the GLU test chip meets the requirements.

## 4. Conclusions

(1) The optimal preparation condition for bifunctional BaSO_4_ sheets under natural drying conditions are as follows: 0.03:1 as CA/BaSO_4_ mass ratio; 2 h as mixing time; 54.9% as solid content and 450 µm as wet sheet thickness.

(2) The reflection spectrometry detection results show that in the wavelength range of 400~800 nm, a bifunctional sheet prepared with BaSO_4_ is from 97% to over 98%, which is obviously higher than that of TiO_2_ bifunctional sheet. GLU-BaSO_4_/CA chip shows wider response range than that of GLU-TiO_2_/CA chip.

(3) The BaSO_4_ bifunctional sheet used in HDL dry chemical in vitro diagnostic reagent shows good repeatability because the spot color is uniform and area is similar which indicates that the serum sample can be diffused uniformly into the reagent sheet.

## Figures and Tables

**Table 1:** Effect of solid content on the surface and flatness of bifunctional sheets.

Acetone Volume (mL)	Solid Content (%)	The Surface and Flatness of Bifunctional Sheet
8	60.1	uneven
10	54.9	uniform and flat
12	50.5	thin and porous

**Table 2:** Relationship between the thickness of wet sheet and scattering power.

Sheets Code	Wet Sheet Thickness (µm)	Scattering Time (s)	Scattering Diameter (cm)
1	200	27	2.3
2	250	20	2.0
3	300	17	1.9
4	350	14	1.6
5	400	12	1.3
6	450	11	1.2
7	500	11	1.2

**Table 3:** Light reflection signal values of dry chemical diagnostic reagents in vitro.

Serial	Single Number (V)	Mean Single Value	Deviation
1	0.3295	0.3250	0.0045
2	0.3259		0.0009
3	0.3238		−0.0012
4	0.3226		−0.0024
5	0.3230		−0.0020

**Table 4:** Repeatability of TiO_2_/CA and BaSO_4_/CA detection chips.

Chip Name	Concentration (mmol·L−1)						SD	CV (%)
GLU-TiO2/CA	8.44	8.81	8.68	8.19	8.27	8.47	0.24	2.8
GLU-BaSO4/CA	8.23	8.19	8.61	8.12	8.54	8.46	0.20	2.4

**Table 5:** Accuracy of BaSO_4_/CA detection chip.

Chip Name	Concentration (mmol·L−1)			Target Value (mmol·L−1)	Range of Target Values (mmol·L−1)
GLU-BaSO4/CA	1.58	1.75	1.72	1.67	1.50~1.84
